# The application of project-based learning in bioinformatics training

**DOI:** 10.1371/journal.pcbi.1005620

**Published:** 2017-08-17

**Authors:** Laura R. Emery, Sarah L. Morgan

**Affiliations:** European Molecular Biology Laboratory, European Bioinformatics Institute, Wellcome Genome Campus, Hinxton, Cambridge, United Kingdom; Genome Quebec, CANADA

This is a *PLOS Computational Biology* Education paper.

## Introduction

As the rate of biological data generation continues to outstrip the rate at which life scientists are able to analyse and add meaning to these data, there is an increasing expectation for an ever more diverse group of life scientists to gain such analytical skills. Providing high-quality training in bioinformatics is more important than ever and, to this end, identifying learning practices and methods that contribute to training success is critical. We present here: the application of project-based learning in the context of a short (5-day) bioinformatics training course, an attempt to assess the impact upon participants of applying such an approach, and guidance to others who might wish to employ such a method in their courses.

Effective bioinformatics training (as in other fields) is recognised as being engaging, promoting active thinking, and providing opportunities for interactivity and discussion. With a plethora of potential learning methods at trainers’ disposal, how do we know which methods are best? The consensus of opinion in the bioinformatics training literature is that diversity is key; by combining a range of approaches, we best meet the needs of a varied participant group [1–3]. Nonetheless, it is likely that some methods will be more effective than others, and thus, here, we explore the application of one such method, project-based learning, in the context of a 5-day training course: a bioinformatics summer school (http://www.ebi.ac.uk/training/events/2014/joint-embl-ebiwellcome-trust-summer-school-bioinformatics-0) that took place in June 2014. The summer school is an established part of a broader training programme offered by EMBL-European Bioinformatics Institute (EMBL-EBI), which includes both face-to-face and elearning opportunities (www.ebi.ac.uk/training).

Project-based learning has long been used in higher education as a method to educate students using realistic problem-based tasks [4,5]. These tasks typically require initiative and independence from the students; they take a considerable length of time to complete, result in the production of an end product (e.g., a report or presentation), and make use of educators in an advisory role [6–8]. Projects can often involve students working together in groups—an approach already adopted as good practice in bioinformatics training [9]—to foster collaboration and develop interpersonal competencies. While there are reports of group-based projects being used in university-level bioinformatics education [10–12] and there are a small number of training courses worldwide that take such an approach (e.g., Cold Spring Harbor’s “Programming for Biology” [13], SFU’s “Problem-based learning in bioinformatics” course for PhD & MSc students [9]), project-based learning is not yet embedded in mainstream bioinformatics training practice. The benefits of using such approaches are clear; however, the process of embedding a project-based approach within such a short course is not a trivial matter.

In redesigning our 2014 summer school to include a project component, we wanted to overcome the challenge of composing a training course that could develop the competencies [14] of an increasingly heterogeneous audience of life scientists. We therefore created a course in which participants with common goals were able to work together using a project-based approach. To assess the impact of applying such an approach, we compared results from course surveys completed by participants: one taken during our previous, traditionally designed course in 2013; a second taken during our redesigned course in which the project-based learning approach was implemented in 2014; and a third completed by the participants who attended our 2014 course 6 months after it had taken place.

## Course design

The joint EMBL-EBI–Wellcome Trust Bioinformatics Summer School is aimed at professional life scientists working at the bench who have little or no experience in bioinformatics but are beginning to work with biological data. Previously, the course focused on a traditional conceptual grounding in a range of bioinformatics topics, with a specific emphasis on sequence searching, alignment, phylogenetics, structural bioinformatics, and networks and pathways. However, with the advent of new technologies and the diversification of the field of bioinformatics, it became increasingly challenging to meet the participants’ varied training needs. With a duration of 5 days, the course cannot cover all essential aspects of bioinformatics. To overcome this challenge in 2014, we redesigned the course to include a substantial project-based learning component. This enabled participants to focus on their specific area of research interest while still being exposed to general principles in bioinformatics and developing the competencies required to conduct sound bioinformatics-based research science. Table 1 illustrates the course design, which includes 2 days of taught theory and general principles, 2 days of project-based learning, and a day of reflection to reinforce learning.

**Table 1:** Overview of programme design.

Day 1	Taught	AM PM	General principles of bioinformatics Study design for bioinformatics and an introduction to genomics
Day 2	Taught	AM PM	Introduction to group projects, functional genomics, and proteins Introduction to networks, pathways, and chemical biology and tools for bioinformatics
Day 3	Research		Participants work on group projects and keynote lecture
Day 4	Research		Participants work on group projects and keynote lecture
Day 5	Reflection		Group presentations and course feedback

### Group projects

The projects were designed based on the assignment of the 30 final participants to specific groups prior to the course taking place. From the pool of more than 70 individuals who applied for the course, we selected participants on the basis of their suitability for the course (using selection criteria listed on our website: www.ebi.ac.uk/training/handson/application). The selected participants were organised into groups on the basis of their shared research interests. We did not have a specific set of projects prepared at this stage and we therefore kept an open mind when assessing common research interests and attempting to group individuals; a different mixture of applicants in a given year could be suited to very different projects than another. From the selection process in 2014, we identified 6 major themes: “causative mutations, variation, and disease”, “medical metagenomics”, “phylogenetics”, “the bioinformatics of a single gene or protein”, “host–pathogen interactions: RNA-Seq and network biology”, and “data management”. We placed 3–6 participants in each of these groups. In our preparation, we recruited and briefed around 3 mentors per project. The mentors were active researchers (predominantly postdoctoral researchers) who had expertise in the project themes and an interest in developing their training skills. They were responsible for both the design and the delivery of the group projects based on their combined knowledge and expertise. We met with the mentors well in advance of the course to ensure that they were familiar with the key principles of project-based learning and to provide guidance on the development and implementation of appropriate projects (Box 1). Further details of the design and implementation of the project-based approach are available in S1 Text.

**Box 1. Key tips for mentors**

- The project should ask interesting research questions and have a realistic biological context relevant for the whole group of participants, based on their research interests and prior experience in computational biology.
- You should provide participants with a starting dataset (or links to appropriate data resources so that they can obtain the dataset), background information about the data and biology, and 2–3 open-ended research objectives.
- Selecting this dataset can be the hardest part of designing a project; it is important to check that it is suitable for meaningful analysis. An ideal dataset, once analysed, allows participants to draw biological conclusions and to answer and ask further questions.
- Remember that you are there to facilitate learning. (Do not complete tasks for the participants!)
- Encourage independent thinking and allow the participants to pursue their own questions.
- You might be asked about things that you don’t know about. If this happens, don’t try to cover this up! Simply direct the participants to someone who is more of an expert in that area than you are.

Course organisers reviewed the projects at a number of stages in development to ensure that each was of appropriate clarity and depth and provided sufficient opportunity for the development of bioinformatics competencies. Mentors were allowed flexibility, and each project was approached in a slightly different manner. This enabled mentors to better cater to the heterogeneous topics on offer and to the differences in the participants’ prior knowledge.

Participants were introduced to the concept of the project during a session on the afternoon of day 1 and were allocated to their groups. They were then introduced to their mentors on day 2 and given the opportunity to start their discussions. Groups then worked through their projects, recording their progress in a shared “lab book” (using Google Docs) that they were able to access post course. Participants worked semi-independently, with mentors providing support when needed. Time on the final day allowed for reflection on the project outcomes through the delivery and critique of group presentations.

## Participant feedback

**Fig 1:**
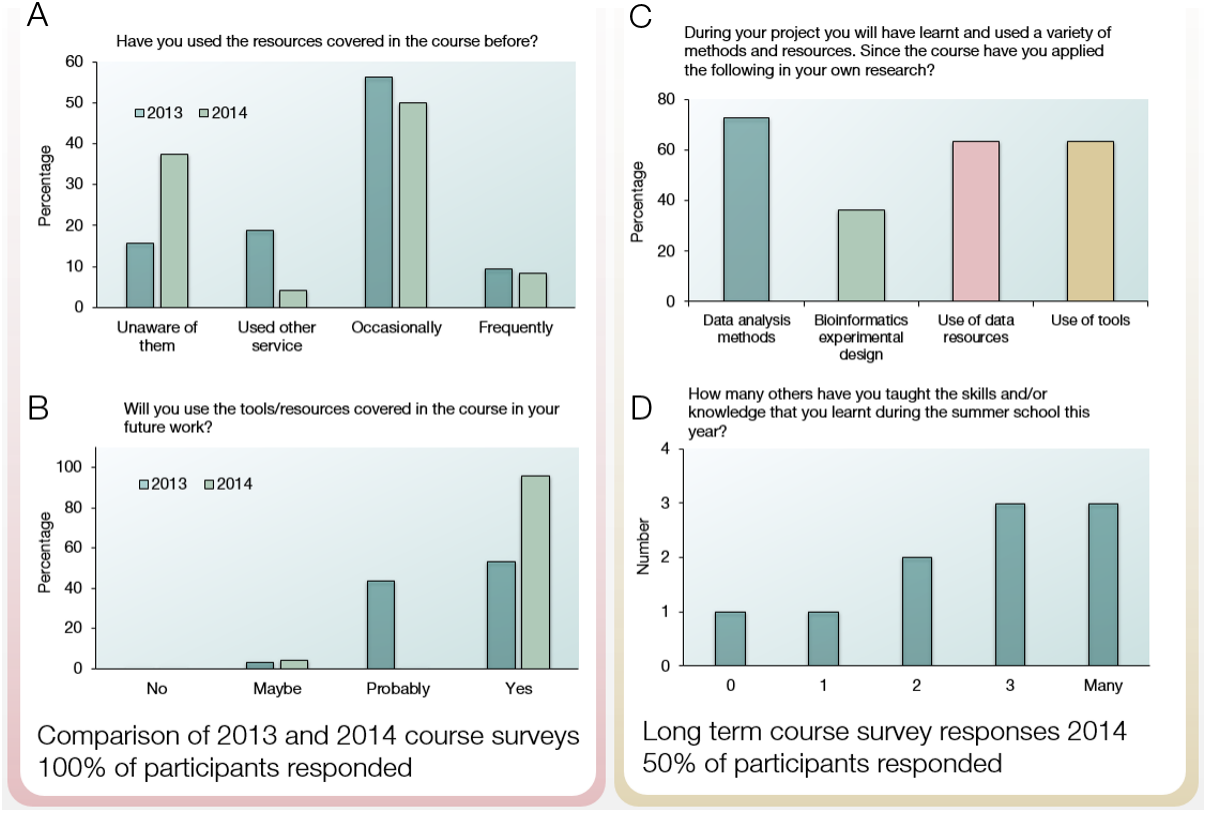
Summary of short- and long-term course surveys.

To assess the success and impact of the training provided, we compared results from 3 post-course surveys: one from the 2013 course based on the original programme, another completed immediately after the redesigned 2014 course, and a third completed by the 2014 cohort 6 months after the course to assess longer-term impact. By comparing the 2013 and 2014 surveys, it is evident that the inclusion of group projects is a popular addition among participants. For example, in the 2014 immediate post-course feedback, “group projects” were cited as “the best part of the course” more frequently than any other comment (see S1 Fig). In 2014, there were also slightly higher levels of course satisfaction, with 100% of participants having stated that they would recommend the course to others, in comparison with 94% in 2013 (data available in S1 Data). It is also interesting that in 2014, a larger proportion of participants were unaware of bioinformatics resources before they attended the course (Fig 1A), suggesting a less experienced cohort. It is therefore striking that after the course, all but a single participant felt confident to use these resources, in comparison with just 53% of the 2013 cohort (Fig 1B).

Immediately post course, many participants from the 2014 cohort left specific comments about the group project expressing their satisfaction. Two such examples are listed below and highlight some of the benefits of the project-based learning approach:

“The group project is an excellent idea, because we can actually visualize and work on real data and learn how to use the tools available to answer the question for the project. The interaction with the tutors was really necessary, as the 3 of us had no previous experience with RNA-Seq data, command line-based tools, or even how to construct and interpret a network of protein interactions. I am pretty sure I will use this on my own work.”

“It was excellent, our team was very interactive, hardworking, and listened to all kinds of ideas and approaches, testing them and having a critical view in the use of different tools. Our mentors gave us the tools that we needed to check our data and our hypothesis. Every time that I was asking how I could approach a search or information using bioinformatics tools, they offered us not just a single one but a variety of tools, explaining to us the advantages or disadvantages between them depending on the problem that be needed to be answered.”

To assess the longer-term impact of the 2014 course, we also sent out a post-course survey to the 2014 cohort 6 months after the course had taken place. This yielded a 50% response rate. The long-term survey showed that the vast majority of participants who attended the 2014 course had subsequently implemented the methods, tools, and resources that they used during the course in their research (Fig 1C). The impact of training can, however, extend far beyond its direct effects on the work of the individuals who attended the course. For example, 2 collaborations were established as a result of the course, which is surprising given the relatively inexperienced cohort who attended. Furthermore, we have seen that a minimum of 14 additional people have been trained directly as a consequence of those attending the course having returned to their labs to train others (Fig 1D). If we also consider the 3 participants who attended the course with responsibilities for teaching undergraduates, MSc, and PhD students, then we see that more than 100 people have been trained as a result of the course. Participants also commented specifically about the advantages of the project-based learning element in the long-term feedback (Box 2).

**Box 2. Complete list of participant comments from the long-term feedback survey, “Please comment on the best aspect of working on the group project this year”**

- The best aspect was to figure out the gene of origin with different tools and discovering and sharing new methodologies to search the genome.
- Very practical: with problem solving, you are forced to critically apply what you learn and therefore test your comprehension.
- It is always good to work as a group because we can unite our skills and knowledge. This makes the process better and faster.
- Being able to communicate with other team members who are from various backgrounds.
- Communication and sharing experiences.
- The good thing is that everyone is learning at the same time and that we can discuss this, so it makes it easier to follow the pipelines for data analysis to get the final result.
- Helps to retain the main concepts of the course a lot better than by lectures only.
- The practical experience in an area directly related to my work has been invaluable. You could have replaced it with ten lectures on the subject and I wouldn’t have learned as much.
- Teamwork and the scientific discussion.
- Good for discussions and for exchange of skills.
- Exchange of visions, ideas, and approaches.

## Discussion

Here we have described the application of project-based learning in the context of the EMBL-EBI–Wellcome Trust Bioinformatics Summer School. We have demonstrated that a project-based approach can be successfully implemented within the scope of a short training course, and key performance indicators from our post-course survey imply that this approach has been more successful than the traditional lecture/tutorial and computer-based practical design used previously. We have also seen that project-based learning impacts positively upon subsequent work undertaken by participants and provides benefit to the extended scientific community.

### The benefits of project-based learning

The qualitative data provided by the participants highlight many of the benefits of project-based learning in the context of bioinformatics training. For example, many participants cite the opportunities for communication, interaction, and discussion with other group members as a positive experience. This supports the views of others, who have noted the benefits of group work in other bioinformatics training contexts [9, 10]. These benefits are further supported in the wider education literature, where it has long been known that co-construction, the collaborative learning process of constructing shared knowledge, can be more effective than direct instruction [4, 6, 7], as multiple members of the group engage with each other to learn more efficiently. The process of co-construction also provides the opportunity for participants to develop relevant interpersonal competencies required for working in a bioinformatics team [14]. The practical nature of the group-project work means that the knowledge, skills, and behaviours learned can be readily applied to other research situations, and the multimodal nature of the learning experience means that projects are well suited to a range of learning styles [15].

The interaction among trainees and mentors was also cited as very helpful. Not only did the mentors design the realistic research scenarios known to be helpful in a bioinformatics training context [16] but they were also present to provide support with both theoretical and practical aspects of the projects, tailoring their support to trainees’ needs for a personalised learning experience. Through discussions, the mentors also had the opportunity to develop participants’ critical thinking skills; a vital competency in computational biology [14]. Some mentors provided more support to participants than others, and while we noticed a slight tendency for these mentors to be more popular among participants during the course, we feel there may be a fine balance to strike between offering groups too much independence versus too much support.

### Our experience

From an initial comparison of our courses, we have found the introduction of project-based learning had a positive impact on the summer school, and we will continue to monitor these benefits as we run additional courses. Developing such an approach is not trivial and required input from a significant number of EMBL-EBI staff and students acting both as trainers and as mentors. Their support has provided impact on several levels: impact on the trainees; increased material available for teaching; and, importantly, an increased group of individuals with training experience beneficial to their future work and to our training programme.

Finally, we would like to offer tips to others who might be thinking about doing the same, based on our experience:

- Projects do not need a defined end point, but initial scope is key to their success; it needs to be wide enough to allow groups to use their own initiative but without the potential for groups to go completely off topic.
- Initial project development is fairly time-consuming but, once defined, a project does have the potential to be reused, with minor revisions as appropriate.
- Providing shared lab notebooks to record details of the project is important for reproducibility and as post-course reference material.
- Mentors should be researchers with broad and current knowledge of theoretical and practical aspects of bioinformatics approaches in their discipline.
- Mentors need to be present in enough numbers (2–3 per group of 3–5 students) and be flexible in their approach to the trainees and the support they provide.
- Providing the opportunity for participants to demonstrate what they have achieved during the project to others external to their group is an important element in their learning path.

We are pleased to say that, owing to the success of this initial course, the format has been applied again in 2015 and 2016.

## Supporting information

S1 Text Provides additional information about the course and project design and implementation. (DOCX)

S1 Data Provides survey data. The first tab (S1 Data S1A) provides survey data from the 2013 post-course survey, the second tab (S1 Data S1B) provides data from the 2014 post-course survey, and the third tab (S1 Data S1C) provides data from the 2014 long-term survey (6 months post course).
(XLSX)

S1 Fig Wordcloud with responses to “What was the best part of the course?” in the 2014 survey. The size of the text indicates the number of occurrences of each word.
(TIF)
