# Supplementary material for: The application of project-based learning in bioinformatics training
Source: PLoS Comput Biol. 2017 Aug 17;13(8):e1005620. doi: 10.1371/journal.pcbi.1005620 (PMC5560525; doi:10.1371/journal.pcbi.1005620)
Supplement: S1 Text — (DOCX) [file pcbi.1005620.s001.docx]

**S1 Text: Additional information**

**1.1 Programme Design**

In 2013, the [course programme](https://www.ebi.ac.uk/training/events/2013/joint-embl-ebiwellcome-trust-summer-school-bioinformatics) was focussed around traditional bioinformatics theory in sequence searching, alignment and phylogenetics, and also included an introduction to the newer topics of functional genomics and protein interactions. In 2014, the [course programme](https://www.ebi.ac.uk/training/events/2014/joint-embl-ebiwellcome-trust-summer-school-bioinformatics-0) was remodelled to provide some initial sessions that were widely applicable to different disciplines within bioinformatics, as well as group projects (S1 Text 1.2) centred on themes relevant to the participant’s research interests. These included ‘study design for bioinformatics’ to cover issues of reproducibility, specificity, sensitivity and how they may be misused; ‘the bioinformatics one-way mirror’ to illustrate how authors often make mistakes in bioinformatics when they forget that the failure to detect a feature in bioinformatics, does not mean it does not exist; and ‘tools for bioinformatics’ which explored the widely applicable tools such as webservices (including those using the command line) that are of use regardless of bioinformatics discipline.

**1.2 Project Planning**

Mentors were recruited 6 months ahead of the course and met several times with the course organisers to receive guidance on how to design a project. Once participant selection had taken place (3-4 months prior to the course), mentors received a copy of the CVs of the participants in their group, so they could ensure that their project design was relevant for all of the students. A large emphasis was placed on designing projects which incorporated open-ended biological research questions. Mentors were required to provide:

A starting dataset (or information to retrieve one)

A project scenario

Project scenarios were descriptions of the project to provide background information for participants. Mentors were advised to keep these short (no more than 2 sides of A4) and to follow a project template, to maintain consistency among projects. The template included three sections: *Scenario*, to provide background information on the biological problem; *Dataset*, to provide details of the dataset they were to use; and *Project aims*, typically three biological questions for the project to focus on, ideally including one that is open-ended so there is scope for extension. Mentors were also provided with additional guidance, including (but not restricted to):

Ensure the project aims to answer a biological question (‘doing X analysis’ is not an aim)

The dataset should be relatively small in size so that it can be processed within the time frame of the course

Remember that what beginner participants can achieve in 2 days is much less than you can achieve in this time, so keep the aims realistic but with scope for further investigation

You are not providing instructions for how to conduct their analyses; this is something the participants may first attempt themselves, and may ask you for informal discussion on during the project

Two weeks prior to the course, project scenarios were reviewed by an organiser. In some cases, projects needed to be amended, and typically this was in cases where mentors had provided details of which analyses they expected students to run, rather than the biological question that underpinned the analysis. An example of a project scenario has been provided [here](ftp://ftp.ebi.ac.uk/pub/training/publications/Group3Metagenomics_outline.pdf).

**1.3 Mentors and project implementation**

Mentors were recruited from the staff at EMBL-EBI; this included some regular trainers, and also many postdocs and a few experienced PhD students. In total there were 18 mentors for the six projects. This is a relatively large number of trainers for a course with 28 participants; however the large number of mentors enabled the workload to be shared and expertise to be combined. Mentors were not required to be present for the duration of the projects; typically one mentor from the group was present and others were on call, thus reducing the workload of any individual mentor. This was made greatly more feasible because we are an institution where a large pool of mentors could be drawn from the staff working on site, and so their contribution on the course did not take them away from their day job for the time that they were not needed. We can envisage that there will be many other institutions that could achieve a similar set up, but acknowledge that there will be some institutions for which this is not possible.

During the group projects, students were given some time to work independently as a group, and at the start of their projects were encouraged to make a plan to go forward. They were, for example, able to decide whether there should be a division of labour of the tasks. A strong emphasis was placed on documenting their progress and the different stages of their work in a shared document known as their ‘group lab book’ ([see example](ftp://ftp.ebi.ac.uk/pub/training/publications/Group3Metagenomics_labbook.pdf)), to ensure that their analysis could be reproduced by any one of them once the course was over. Mentors provided the level of support that they felt was most beneficial for the group. This typically included introducing the project, giving the students space for planning, and assisting them when they got into difficulty and responding to their questions. Additionally mentors provided informal project-specific theory to covey key concepts.

After the course, a meeting was arranged with the mentors to discuss their experiences. The consensus of opinion was that they had enjoyed running the projects; for many it was the very first time they had trained, and for others it was the first time they had trained as a mentor. Some of these first time trainers have now become regular trainers on our other courses, and so it has been successful in providing a new route into becoming a trainer. Most acknowledged that designing the project itself was a lot of work but this did not seem to put them off from volunteering in subsequent years. In 2015, 13 of the 18 mentors returned, which represents a high level of commitment.

**1.4 Group Presentations**

On the afternoon of the second day of project work, students were encouraged to prepare short (7 minute) group presentations for the following morning. The medium they chose to present in was up to them but we asked them to include:

Brief outline of project and its aims

Description of analyses, tools and workflows used

Results

Discussion

The act of producing a presentation enabled the students to review and consolidate their work. The presentations took place on the morning of the final day of the course, and included challenging questions from mentors and instructors, and so was also a useful opportunity for the students to develop their skills in presenting and debating scientific work. In viewing the presentations from other groups, they had the opportunity to develop their scientific questioning, as well as gaining an awareness of other areas of bioinformatics not covered in their projects.

**1.5 Compute setup**

No special compute requirements were needed for this course because we encouraged mentors to design projects with small datasets and so all could be conducted on standard desktop PCs. Mentors requested relevant software to be installed prior to the course, and there was also time to install additional software during the course on an adhoc basis.

**1.6 Survey collection**

We always take course surveys on the last day of our courses to ensure we have a good response rate. For example in 2013, 100% (32/32) participants answered the survey, and in 2014, 86% (24/28) participants answered the survey. By definition, the long-term survey feedback from 2014 had to be collected post-course, and so the lower response rate of 50% reflected this. Nonetheless a 50% response rate would be considered high with surveys in general, where the typical industry standard is a 5% response rate. Other long-term feedback in bioinformatics training has yielded a 25% response rate (Gabriella Rustici, personal communication).
