# Supplementary figures and images for: The application of project-based learning in bioinformatics training
Source: PLoS Comput Biol. 2017 Aug 17;13(8):e1005620. doi: 10.1371/journal.pcbi.1005620 (PMC5560525; doi:10.1371/journal.pcbi.1005620)

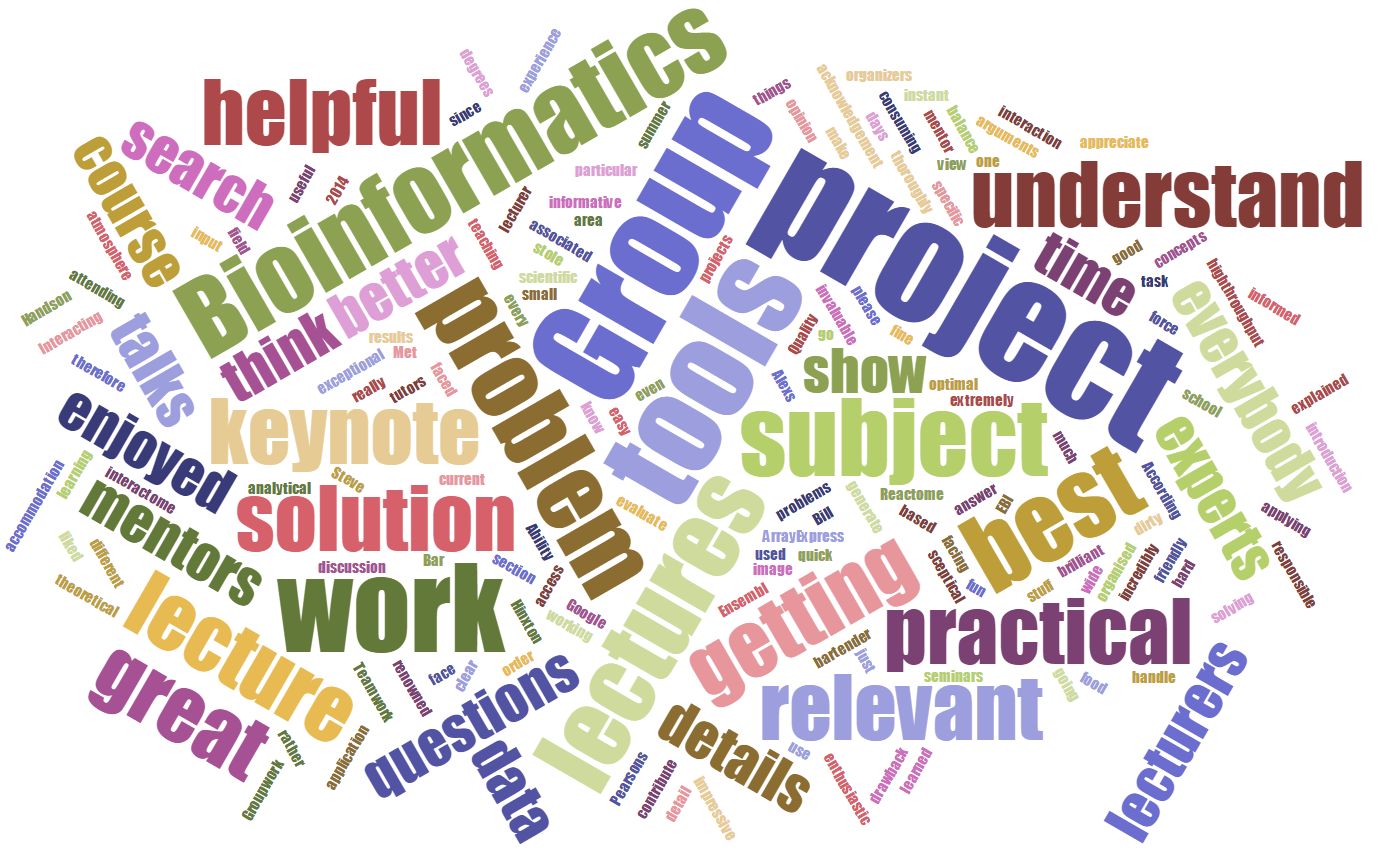

Supplement: S1 Fig — The size of the text indicates the number of occurrences of each word. (TIF) [file pcbi.1005620.s003.tif]
